# Supplementary material for: Image-Based Methods to Score Fungal Pathogen Symptom Progression and Severity in Excised Arabidopsis Leaves
Source: Plants (Basel). 2021 Jan 15;10(1):158. doi: 10.3390/plants10010158 (PMC7830641; doi:10.3390/plants10010158)
Supplement: Supplementary file 1 [file plants-10-00158-s001.pdf]

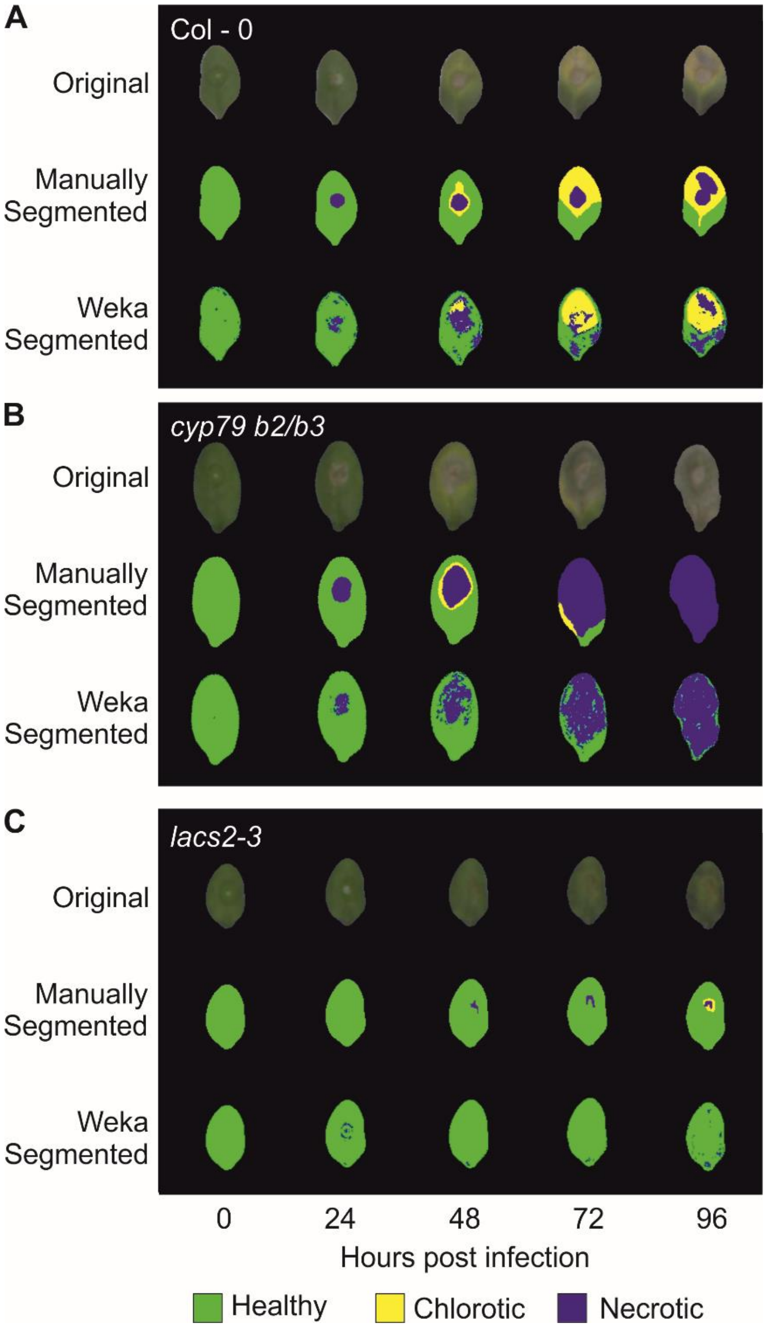

Figure S1. Comparisons among manually and weka-segmented Arabidopsis leaves. (**A**) Col-0, wild type Columbia-0 accession; (**B**) *cyp79 b2/b3*, the Botrytis-susceptible *cytochrome p450 79-b2* and *-b3* double mutant; (**C**) *lacs2-3*, the Botrytis-resistant *long-chain acyl-coa synthase2* mutant. Green, healthy pixels; yellow, chlorotic pixels; purple, necrotic pixels.

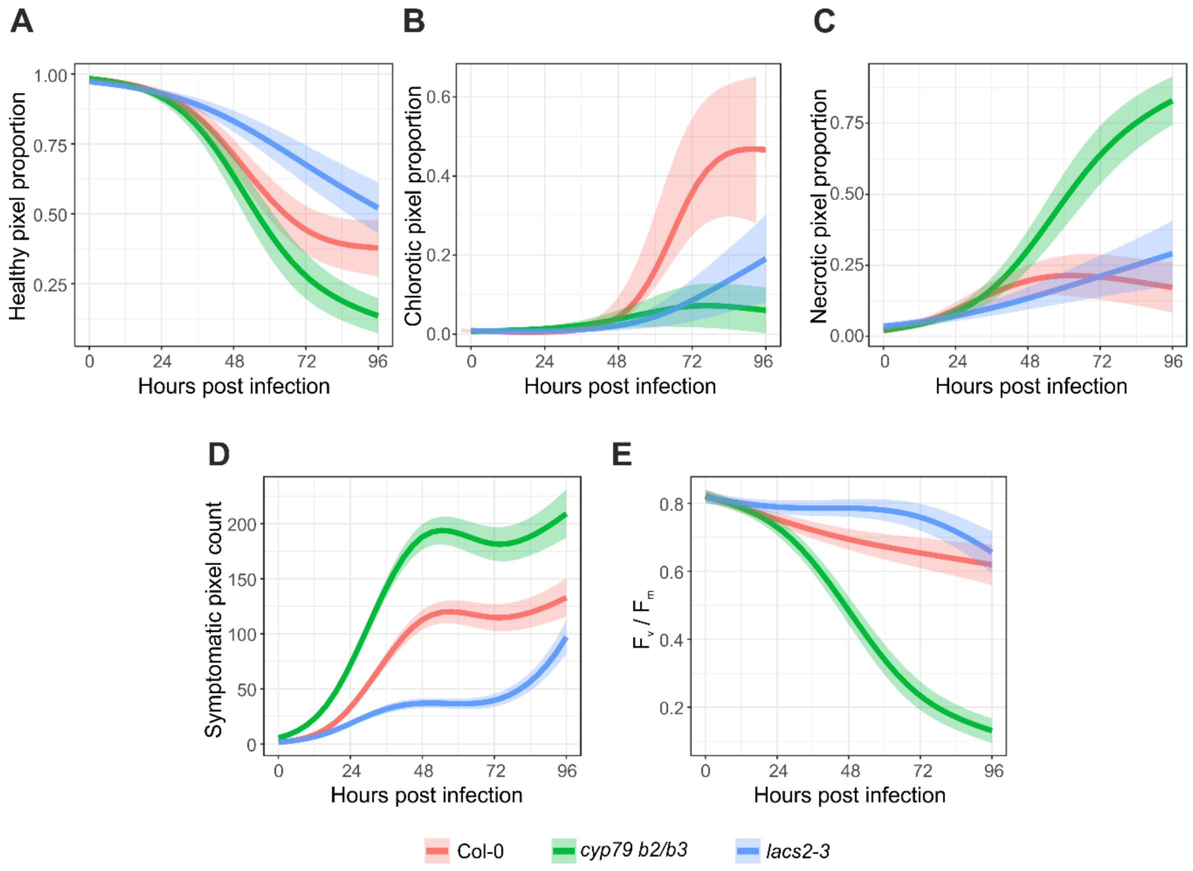

Figure S2. Model fit for all parameters used in this study. (**A**) Healthy pixel proportion; (**B**) Chlorotic pixel proportion; (**C**) Necrotic pixel proportion of the total pixels in the leaf; (**D**) Symptomatic area estimated by counting all pixels with F_v/F_m values below 0.75; (**E**) Maximum quantum yield of photosystem II was used as a severity measurement. (A–C) used red green blue (RGB) imaging and (D,E) used chlorophyll fluorescence imaging. Col-0, wild type Columbia-0 accession; *cyp79 b2/b3*, the Botrytis-susceptible *cytochrome p450 79-b2* and *-b3* double mutant; *lacs2-3*, the Botrytis-resistant *long-chain acyl-coa synthase2* mutant; F_v/F_m, maximum quantum yield of photosystem II or severity.
